# Editorial for Special Issue “Diagnosis and Treatment of Cervical Cancer”

**DOI:** 10.3390/medicina61061057

**Published:** 2025-06-09

**Authors:** Giorgia Perniola, Tullio Golia D’Augè

**Affiliations:** Department of Maternal and Child Health and Urological Sciences, Sapienza University of Rome, Policlinico Umberto I, 00161 Rome, Italy; giorgia.perniola@uniroma1.it

Cervical cancer remains one of the most significant gynecologic malignancies worldwide, particularly impacting women in their reproductive years [1]. Despite the widespread use of HPV vaccination and cervical screening programs, the disease burden remains high, especially in low-resource settings [2,3]. Advances in diagnostic technologies, conservative management strategies, and tailored surgical approaches have modified the clinical landscape, aiming to improve oncologic outcomes while preserving fertility and quality of life in affected patients [4,5,6,7,8].

This Special Issue features five original contributions that address the present critical challenges in the prevention, diagnosis, and treatment of cervical intraepithelial neoplasia (CIN) and early cervical cancer (CC). These works highlight the gynecologic community’s continued commitment to refining conservative and surgical approaches in order to improve oncological outcomes and preserve reproductive potential.

The retrospective study by Ferrari et al. compared cold knife (CK) and CO_2_ laser conization for preinvasive cervical lesions, showing a significantly lower rate of positive endocervical or deep margins with CO_2_ laser [9]. Notably, both techniques demonstrated oncological safety in cases of incidental early-stage cervical cancer, highlighting their potential role in fertility-sparing treatment strategies.

A comprehensive systematic review by D’Amato et al. analyzed the reproductive outcomes of fertility-sparing treatments (FSTs) in women with tumors >2 cm [10]. Although a meta-analysis could not be performed due to significant data heterogeneity, the authors emphasized a consistent correlation between oncological and reproductive outcomes according to the surgical approach, underscoring the need for future evidence-based recommendations in this specific patient subgroup.

Rotar et al. reviewed the topical treatment landscape for CIN, emphasizing the potential of locally administered agents as alternatives to surgery in selected cases [11]. Their findings underscore the growing interest in conservative therapies aimed at reducing obstetrical complications associated with demolitive procedures.

The prospective observational study by Vitkauskaite et al. explored the role of inflammation in cervical cancer pathogenesis and staging [12]. By analyzing serum concentrations of IL-6, TREM-1, and LCN2 in patients with cervical cancer compared to healthy controls, the authors recognized these markers as independent predictors of the diagnosis of cervical cancer and advanced-stage disease. Their findings suggest the potential utility of systemic inflammatory mediators as cost-effective tools for diagnosis and risk stratification in cervical cancer patients.

Finally, Dicu-Andreescu et al. conducted a retrospective–prospective study on a cohort of 96 patients with cervical cancer stages IA2 to IIIB, treated with neoadjuvant chemo-radiotherapy followed by radical surgery [13]. The authors investigated FIGO stage, residual tumor, stromal and lymphovascular invasion, and resection margin status as patient-related characteristics affecting 3-year survival. Their findings showed that positive resection margins and post-radiotherapy FIGO stage III were independent predictors of modest outcomes. In patients treated with neoadjuvant therapy, the high rate of residual tumor at surgery highlights the importance of tailoring surgical strategies—even in advanced disease—according to individual patient characteristics.

Collectively, these contributions advance our understanding of cervical pathology management, offering new perspectives for clinicians striving to balance oncologic radicality and fertility preservation. We hope this Special Issue will inspire further research and guide future clinical practice in the evolving field of gynecologic oncology.

## Abbreviations

The following abbreviations are used in this manuscript:

CIN	Cervical intraepithelial neoplasia
CC	Cervical cancer
CK	Cold knife
FST	Fertility-sparing treatments
HPV	Human papilloma virus
